# Supplementary material for: Selective recruitment of different Ca2+-dependent transcription factors by STIM1-Orai1 channel clusters
Source: Nat Commun. 2019 Jun 7;10:2516. doi: 10.1038/s41467-019-10329-3 (PMC6555828; doi:10.1038/s41467-019-10329-3)

Supplemental Figure 1 (related to Figure 1).
Stores do not refill in the presence of 30 nM thapsigargin. A, Trace shows cytosolic Ca^{2+} signal to challenge with 30 nM thapsigargin in the continuous presence of external Ca^{2+} (2 mM). After 40 minutes, cells were perfused with Ca^{2+}-free solution (containing thapsigargin) and then ionomycin (2 μM) was added as shown. Control trace shows the response over the same time period but without thapsigargin stimulation. 30 nM thapsigargin trace is the average of 42 cells and control 36 cells. B, Aggregate data measuring the ionomycin response from experiments in panel A are compared. The $0Ca^{2+}$ bar denotes cells challenged with 30 nM thapsigargin in Ca^{2+}-free external solution for 40 minutes and then exposed to ionomycin. Responses were normalised to control.

Supplemental Figure 2 (related to Figure 1).
Ca^{2+} signals evoked by different concentrations of thapsigargin in the continuous presence of external Ca^{2+} are compared. The traces show the cytosolic Ca^{2+} signals over a 40 minutes period. Each trace is the mean of >30 cells.

Supplemental Figure 3 (related to Figure 3). Binomial distribution to characterize fluorescence data. A, The same fluorescence data shown in Fig 3C for 2μM thapsigargin, on a nuclear:cytoplasmic ratio scale. This is transformed using the simple expression shown to a nuclear proportion (assuming nuclear + cytoplasmic = 1). B, the histogram for the same data is shown on the Nuclear Proportion scale with an overlaid Probability Density Function fitted using a Binomial distribution with properties ~Binomial(N=25,p=0.8205). C, Properties of a Binomial distribution. The spread is a function of the number of agents (25) and probability of nuclear translocation (p), with maximum spread for p=0.5. D, The variance of the binomial distributions described by ~Binomial(N=25,p) (square of standard deviation) plotted as a function of 'p' – the mean proportion that translocate to the nucleus. Here the variance of the blue curve shown in Figure 3E for 30 nM thapsigargin is shown alongsidethat of the data, assuming that the probability distribution for this concentration is built from 0.8*Binomial(25, p at Rest) + 0.2*Binomial(25, p at 2 μM thapsigargin).

Supplemental Figure 4 (related to Figure 3). Histograms compare rate of Ca^{2+} entry for the conditions indicated. Cells were stimulated with thapsigargin in Ca^{2+}-free solution for 10 minutes and then external Ca^{2+} (2 mM) was readmitted. The rate of Ca^{2+} entry was measured by differentiating the rise of the Ca^{2+} signal upon Ca^{2+} readmission. Basal denotes the background Ca^{2+} entry rate, obtained by exposing cells to Ca^{2+}-free solution for 10 minutes but without thapsigargin. Note the absence of a binomial distribution for groups challenged with thapsigargin.

Supplemental Figure 5 (related to Figure 4). Effects of different concentrations of thapsigargin on nuclear translocation of STAT5-GFP. A, Images compare

STAT5-GFP distribution for the conditions shown. B, Aggregate data from experiments as in panel A are comapred. Each bar is the mean of >12 cells.

Supplemental Figure 6 (related to Figure 7). Ionomycin restores full NFAT activation in response to thapsigargin/0Ca^{2+}/La^{3+}. Bar chart compares NFAT nuclear accumulation following stimulation with thapsigargin in 2 mM external Ca^{2+} and thapsigargin/0Ca^{2+}/La^{3+} (both for 60 minutes). Application of ionomycin (5 μM) in 2 mM external Ca^{2+} solution (still in the presence of thapsigargin and La^{3+}) for 20 minutes after cells had been challenged with thapsigargin in Ca^{2+}-free solution containing La^{3+} for 40 minutes increased NFAT nuclear accumulation to levels similar to those seen when cells were stimulated with thapsigargin in 2 mM external Ca^{2+}. Each bar is the mean of > 10 cells.

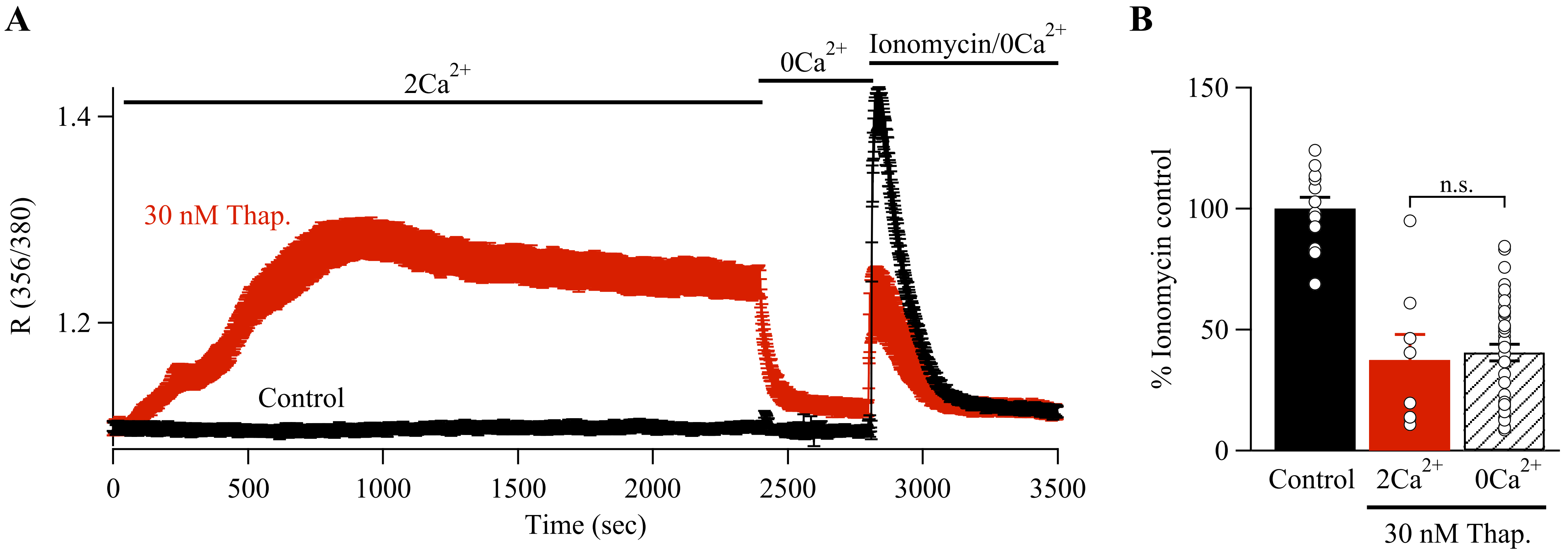
A
2Ca2+
0Ca2+
Ionomycin/0Ca2+
1.4
1.2
R (356/380)
30 nM Thap.
Control
0
500
1000
1500
2000
2500
3000
3500
Time (sec)
B
150
100
50
0
% Ionomycin control
n.s.
Control
2Ca2+
0Ca2+
30 nM Thap.

Supplemental Figure 2

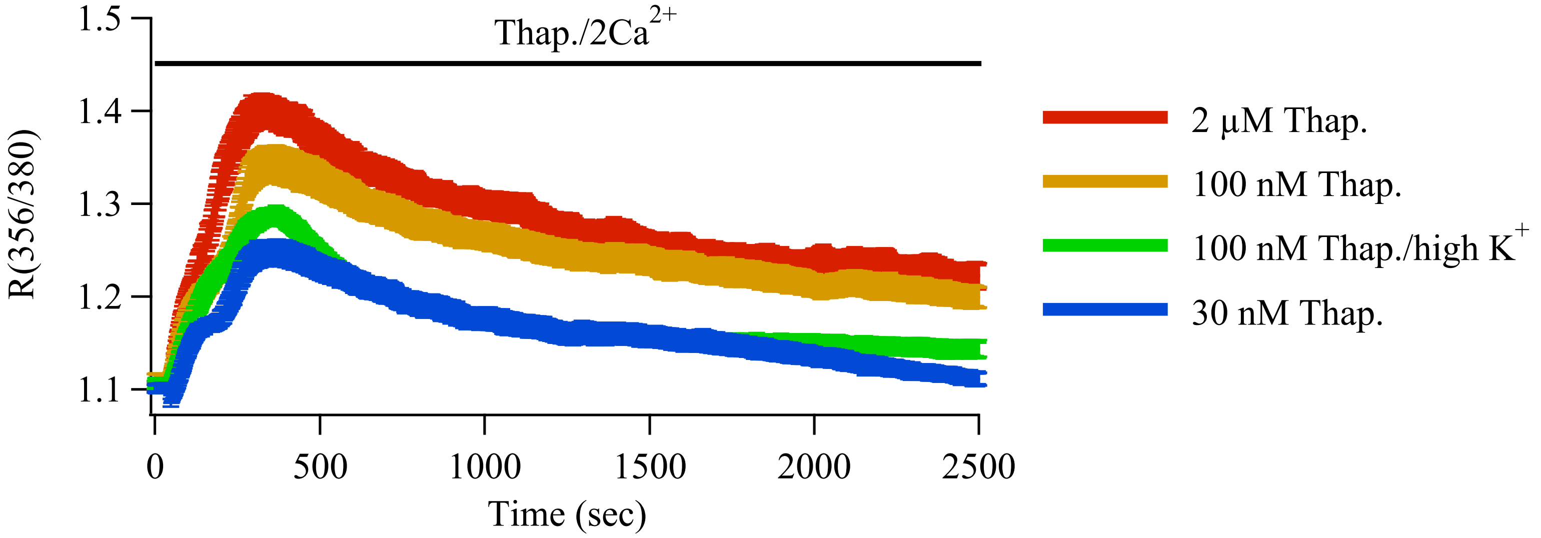

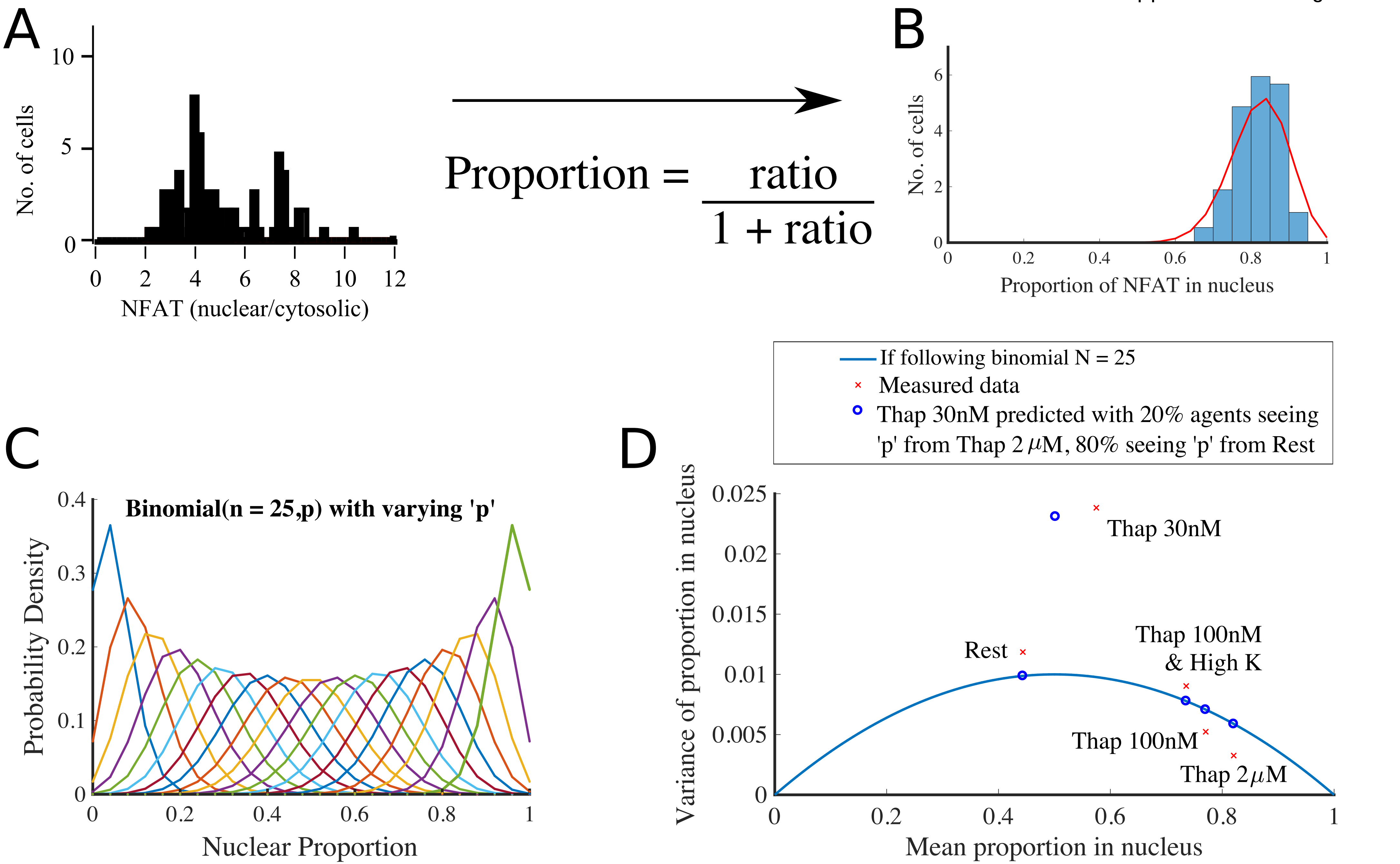
A
No. of cells
10
5
0
0
2
4
6
8
10
12
NFAT (nuclear/cytosolic)
Proportion = ratio / 1 + ratio
B
No. of cells
6
4
2
0
0
0.2
0.4
0.6
0.8
1
Proportion of NFAT in nucleus
If following binomial N = 25
Measured data
Thap 30nM predicted with 20% agents seeing 'p' from Thap 2 μM, 80% seeing 'p' from Rest
C
Binomial(n = 25,p) with varying 'p'
Probability Density
0.4
0.3
0.2
0.1
0
0
0.2
0.4
0.6
0.8
1
Nuclear Proportion
D
Variance of proportion in nucleus
0.025
0.02
0.015
0.01
0.005
0
Thap 30nM
Rest
Thap 100nM & High K
Thap 100nM
Thap 2μM
0
0.2
0.4
0.6
0.8
1
Mean proportion in nucleus

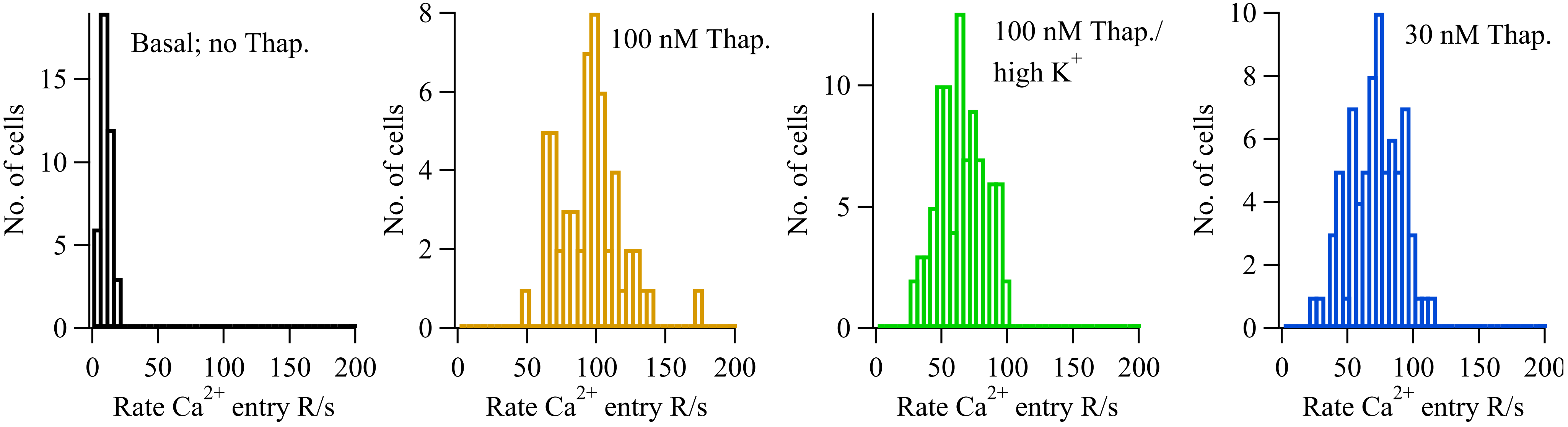
Basal; no Thap.
100 nM Thap.
100 nM Thap./
high K$^+$
30 nM Thap.
No. of cells
Rate Ca^{2+} entry R/s
0
5
10
15
2
4
6
8
50
100
150
200

Supplemental Figure 5

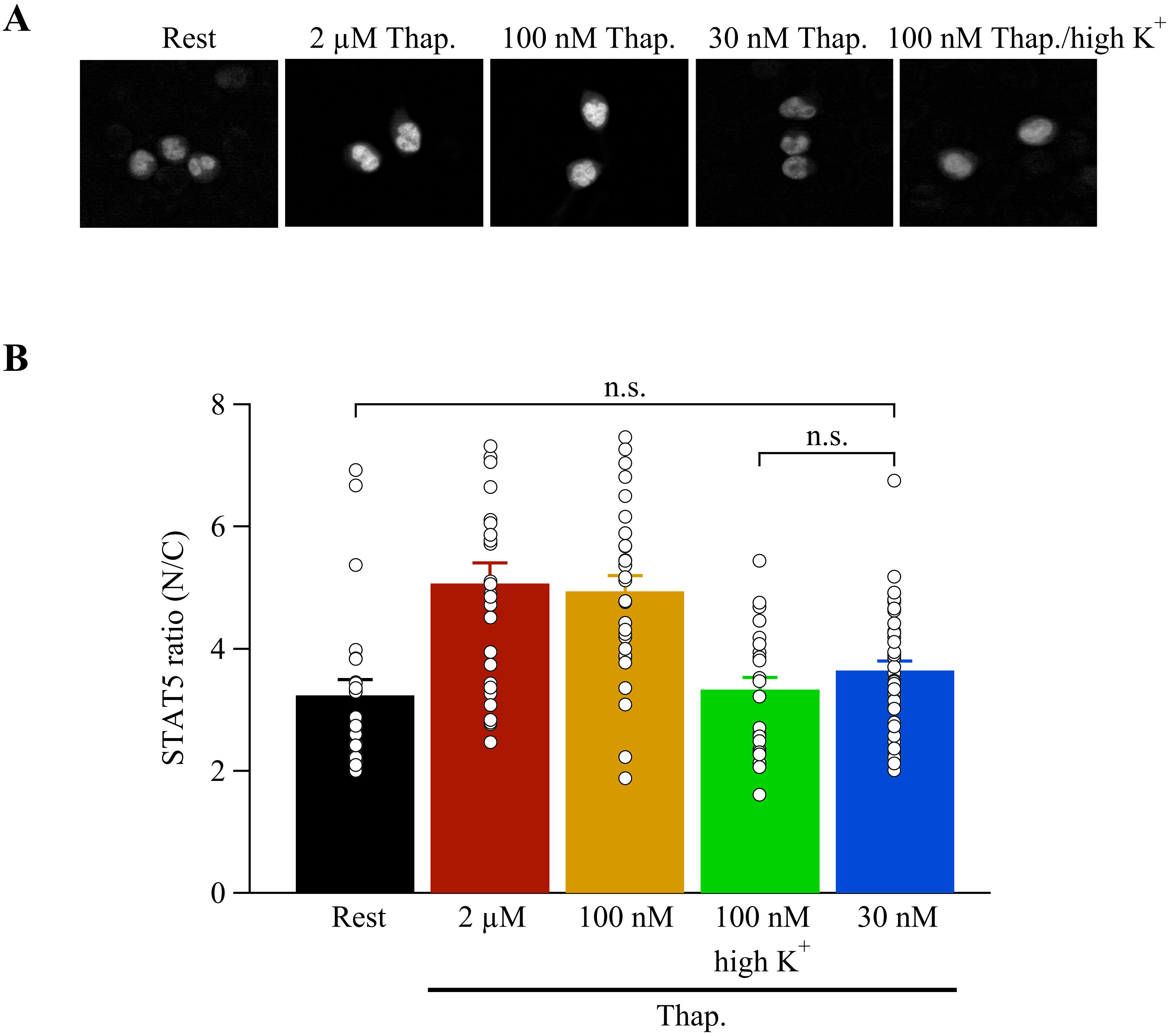

Supplemental Figure 6

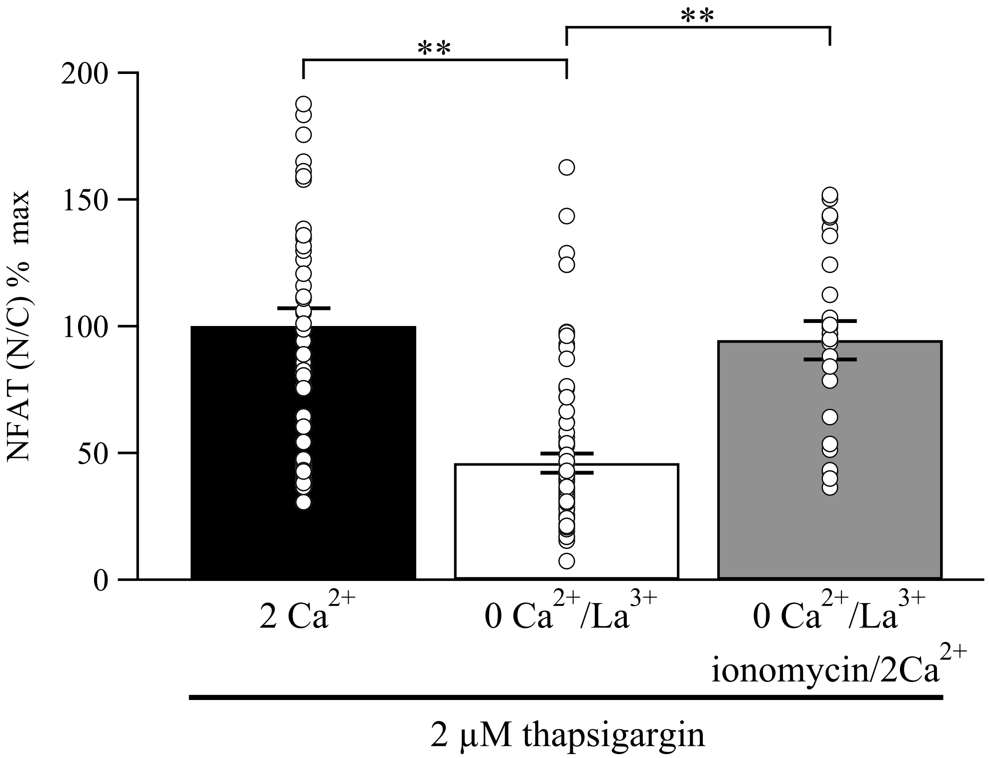

Supplement: Supplementary file 1 — Supplementary Information [file 41467_2019_10329_MOESM1_ESM.pdf]
